# Atrial fibrillation is independently associated with impaired myocardial perfusion

**DOI:** 10.1371/journal.pone.0361402

**Published:** 2026-10-27

**Authors:** Niklas Vartiainen, Suvi Hartikainen, Antti-Pekka E. Rissanen, Elina Kariniemi, Aleksi J. Leikas, Eero Strengell, Svantte Halttunen, Paavo-Ilari Kuikka, Juha E. K. Hartikainen, Tomi P. Laitinen

**Affiliations:** 1 Department of Clinical Physiology and Nuclear Medicine, Kuopio University Hospital, Kuopio, North Savo, Finland; 2 Institute of Clinical Medicine, University of Eastern Finland, Kuopio, North Savo, Finland; 3 Helsinki Sports and Exercise Medicine Clinic (HULA), Foundation for Sports and Exercise Medicine, Helsinki, Uusimaa, Finland; 4 Sports and Exercise Medicine, Faculty of Medicine, University of Helsinki, Helsinki, Uusimaa, Finland; 5 Department of Medicine, Kuopio University Hospital, Kuopio, North Savo, Finland; 6 Department of Clinical Radiology, Kuopio University Hospital, Kuopio, North Savo, Finland; 7 A. I. Virtanen Institute for Molecular Sciences, University of Eastern Finland, Kuopio, North Savo, Finland; 8 Heart Center, Kuopio University Hospital, Kuopio, North Savo, Finland; 9 Gene Therapy Unit, Kuopio University Hospital, Kuopio, North Savo, Finland; 10 Department of Clinical Physiology and Nuclear Medicine, Mikkeli Central Hospital, Mikkeli, South Savo, Finland; 11 Department of Clinical Physiology and Nuclear Medicine, North Karelia Central Hospital, Joensuu, North Karelia, Finland; Emory University, UNITED STATES OF AMERICA

## Abstract

A characteristic feature of obstructive coronary artery disease (CAD) is impaired myocardial blood flow (MBF). Previous studies have shown that atrial fibrillation (AF) is also associated with reduced hyperemic MBF. However, it is unknown whether an independent association exists between AF and MBF in symptomatic patients with suspected obstructive CAD. We studied 874 patients at Kuopio University Hospital (Finland) who underwent coronary computed tomography angiography (CCTA) and [^15^O]H_2_O myocardial perfusion positron emission tomography due to suspected obstructive CAD in 2017–2023. The final cohort comprised 31 AF patients and 120 propensity score-matched sinus rhythm (SR) patients. The matching criteria were age, sex, body mass index, number of traditional risk factors for CAD (0–5), calcium score, and total CCTA status (i.e., the overall severity of CCTA findings). Global hyperemic MBF was lower in AF patients than in SR patients (median 1.9 mL/g/min [IQR 1.4–2.6 mL/g/min] vs. 3.0 mL/g/min [IQR 2.4–3.6 mL/g/min], p < 0.001). Impaired hyperemic MBF (< 2.3 mL/g/min in ≥ 1 segment) was more frequent in AF patients (n = 25/31, 81%) than in SR patients (n = 50/120, 42%, p < 0.001). Furthermore, the presence of AF was independently associated with reduced hyperemic MBF in three multivariate general linear models (p < 0.001 in each model). To summarize, AF was independently associated with impaired hyperemic MBF in patients with suspected obstructive CAD. This association should be considered in clinical practice when interpreting the results of myocardial perfusion imaging in patients with AF.

## Introduction

Despite advances in coronary artery disease (CAD) management, it is still a considerable health burden globally [1]. In recent years, the primary diagnostic methods of CAD have changed. For the initial diagnosis of CAD, the latest American and European guidelines recommend noninvasive imaging in most scenarios [2,3]. Among these methods, coronary computed tomography angiography (CCTA) has emerged as a widely used and practical modality. CCTA enables direct visualization of epicardial coronary arteries and shows the distribution and severity of coronary atherosclerosis. Due to its high sensitivity and negative predictive value, CCTA excels in ruling out CAD even in patients with high pretest probability [4]. CCTA demonstrates lower specificity for confirming obstructive CAD, however [4]. To increase diagnostic accuracy, CCTA can be combined with myocardial perfusion imaging (MPI). Among the various MPI modalities, positron emission tomography (PET) with [^15^O]H_2_O is considered the reference standard for absolute quantification of myocardial blood flow (MBF) [5]. However, impaired MBF is not specific to obstructed epicardial vessels. Instead, [^15^O]H_2_O PET MPI also reflects the perfusion of the coronary microvasculature [6].

Besides CAD, atrial fibrillation (AF), the most common sustained arrhythmia, causes significant morbidity, hospitalization, and mortality [7]. CAD and AF share risk factors and, additionally, CAD is a risk factor for the development of AF [8,9]. Even patients without CAD may present with symptoms and clinical findings suggesting myocardial ischemia if they have AF [10,11]. This may relate to several mechanisms including coronary microvascular dysfunction [12]. Previous studies in various patient populations have reported that when compared to patients with sinus rhythm (SR), those with AF have lower myocardial perfusion [13–18]. Still, whether AF is independently associated with myocardial perfusion in patients referred to diagnostic imaging due to suspected obstructive CAD remains unknown. Also, when investigating the association between AF and myocardial perfusion, previous studies have not utilized the severity and extent of coronary atherosclerosis as confounders.

We studied whether AF is independently associated with impaired myocardial perfusion in patients referred to noninvasive coronary imaging due to symptoms suggesting the presence of obstructive CAD. To investigate this possible independent association, we compared hyperemic MBF in patients with AF and in comprehensively matched patients with SR.

## Methods

### Subjects

We conducted a retrospective, registry-based study of patients who underwent CCTA and consecutive [^15^O]H_2_O PET MPI at Kuopio University Hospital, Finland, from 2017 to 2023. Each patient was clinically referred to the imaging due to symptoms suggesting obstructive CAD. The initial study population comprised 971 patients. Our exclusion criteria encompassed technically unsuccessful CCTA or PET MPI, prior myocardial infarction or coronary revascularization, heart failure, cardiomyopathy, at least moderate valvular heart disease, pacemaker, or any cardiac rhythm other than AF or SR during PET MPI. After applying the exclusion criteria, our final study population included 874 patients. Of these, 31 had ongoing AF, and 843 had SR during PET MPI. Eventually, we used propensity score matching (see the section Statistical methods for details) to match the 31 AF patients with 120 SR patients and included those 151 patients in this study’s final analyses. Fig 1 presents a flow chart for patient selection.

**Fig 1:**
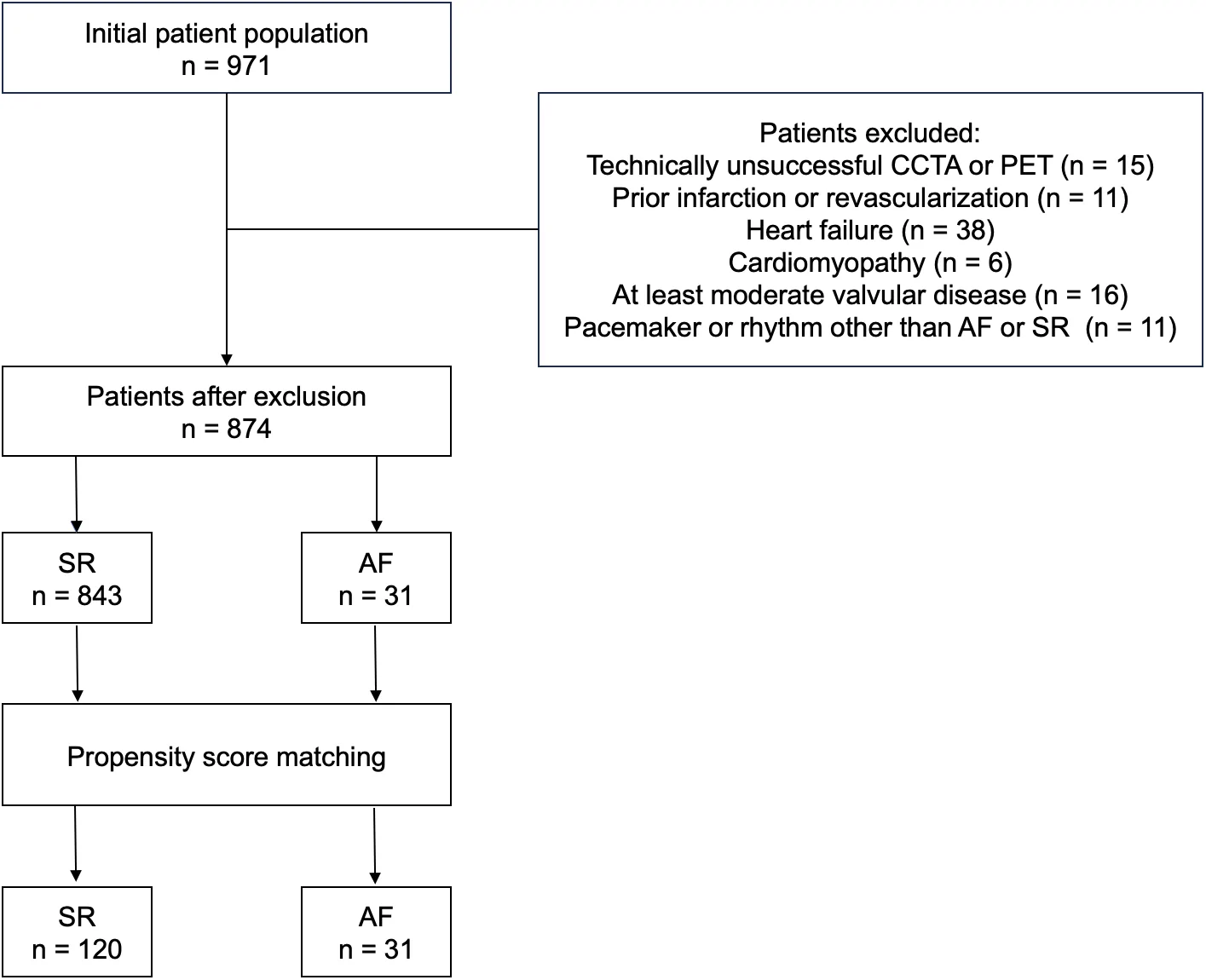
Flow chart for patient selection. AF = atrial fibrillation, CCTA = coronary computed tomography angiography, PET = positron emission tomography, SR = sinus rhythm.

For this research, we secured written authorization from the registrar (the Wellbeing Services County of North Savo) to utilize their data. In Finland, scientific research based only on registry data requires no approval of an ethics committee or informed consent from patients. Therefore, before this study, we received an exemption from ethical approval from the ethics committee of the University of Eastern Finland (description number 16/2023). Data collection took place between February 2024 and September 2024, during which the authors had access to information that could identify individual patients of the study. After the data were collected and analyzed, they were anonymized so that no individual patients could be identified.

### Clinical data

Clinical data were collected from electronic patient records and a questionnaire completed by the patients before CCTA and PET MPI. The data included age, sex, body mass index, medication, angina symptoms, comorbidities, and traditional risk factors for CAD (hypertension, dyslipidemia, diabetes, smoking history, and family history of CAD). Blood test results within three months of PET MPI were collected when available. Ongoing cardiac rhythm was determined based on 12-lead electrocardiography obtained during PET MPI. The type of AF was classified based on electronic patient records.

### Imaging protocol

At Kuopio University Hospital, CCTA serves as the primary noninvasive modality for diagnosing CAD in patients with a low-to-moderate pretest probability. If CCTA is suggestive of obstructive CAD (≥ 50% luminal stenosis in any epicardial coronary artery) or if obstructive CAD cannot be excluded due to suboptimal image quality, patients are referred to PET MPI. Before PET MPI, the patients are instructed to abstain from smoking for at least 4 hours, caffeine for at least 12 hours, and alcohol for at least 48 hours. Also, dipyridamole is discontinued for a minimum of 48 hours before PET MPI. Most patients undergo CCTA and PET MPI consecutively on the same day. Rarely due to scheduling issues or inadequate patient preparation, CCTA and PET MPI are performed on separate days.

### Coronary computed tomography angiography

For this current study, three different CT scanners by Siemens (Siemens Healthineers, Erlangen, Germany) were used for CCTA: Somatom Definition Flash, Somatom Force, and Somatom Edge. If the patient’s heart rate was over 65 beats/min, they were administered up to 20 mg of intravenous metoprolol. Immediately before imaging, the patients also received 1.25 mg of sublingual isosorbide dinitrate.

CCTA imaging began with the acquisition of unenhanced CT images, from which the coronary artery calcium score was determined according to Agatston *et al*. [19]. Next, contrast-enhanced CCTA scans were performed using prospective electrocardiograph-gated acquisition whenever possible. From CCTA images, we determined findings from the left main (LM), left anterior descending (LAD), left circumflex (LCX), and right (RCA) coronary arteries. A status was categorized for each artery: 0 = vessel not interpretable, 1 = no visible atherosclerosis, 2 = calcified or non-calcified plaque(s) with luminal stenosis < 50%, 3 = calcified plaque(s) with luminal stenosis ≥ 50%, 4 = non-calcified or partially calcified plaque(s) with luminal stenosis ≥ 50%, and 5 = total occlusion. This grading was adapted from the 2022 Coronary Artery Disease-Reporting and Data System [20]. A total CCTA status of 0–5 was formed based on the highest status from any individual coronary artery. For example, if a patient’s status for RCA, LM, and LAD was 2, and the status for LCX was 4, the total CCTA status was 4.

### Myocardial perfusion imaging

PET MPI was performed with [^15^O]H_2_O using either Biograph mCT or Biograph Vision PET/CT scanner (Siemens Healthineers, Erlangen, Germany). Imaging was performed during pharmacologically induced hyperemia, achieved with a continuous intravenous infusion of adenosine at a rate of 140 µg/kg/min over 6.5 minutes. During adenosine infusion, an intravenous bolus of [^15^O]H_2_O with a weight adjusted activity of 500–800 MBq was injected, and dynamic images were acquired over 5 minutes. A low-dose CT was used for attenuation correction. Since our standard clinical protocol included only hyperemic imaging, rest images were unavailable for most patients and were excluded from this study. Blood pressure (BP) was measured at rest and immediately after the [^15^O]H_2_O bolus with an automated sphygmomanometer device (SunTech Tango M2, SunTech Medical Inc., Morrisville, North Carolina, USA). A 12-lead electrocardiogram was monitored using the GE Case (General Electric Company, Boston, Massachusetts, USA). Rate pressure product (RPP) was calculated as systolic BP multiplied by heart rate (HR).

PET images acquired with Biograph mCT were reconstructed with two iterations and 21 subsets, with a 3.18 mm x 3.18 mm pixel size. For images acquired with Biograph Vision, image reconstruction was performed with eight iterations, five subsets, and a pixel size of 2.83 mm x 2.83 mm. A matrix size of 128 x 128 and Gaussian 6 mm post-filtering were used for all images regardless of the PET/CT scanner.

Hyperemic MBF was quantified with Carimas software (Turku PET Centre, Turku, Finland) using a single-tissue compartment model [21,22]. MBF was quantified using the standard 17-segment model [23], with measurements recorded for each segment in mL/g/min. MBF was also assessed as a global value and for the standardized perfusion territories of LAD, LCX, and RCA. MBF was considered impaired when any individual segment (excluding basal septal segments 2 and 3) had a value of < 2.3 mL/g/min [24].

### Invasive coronary angiography and revascularization

Based on the noninvasive imaging protocol and patient-specific evaluation, attending cardiologists considered whether to refer individual patients to ICA. Additionally, if significant stenoses were present in ICA, they decided whether to perform revascularization procedures.

### Statistical methods

We formed two groups for statistical analyses: patients who, during PET MPI, had ongoing AF and those who had ongoing SR. To assess the possible independent association between AF and myocardial perfusion, we formed the groups using propensity score matching. Matching was based on six criteria: age, sex, body mass index, number of traditional risk factors for CAD (0–5), calcium score, and total CCTA status [24–26].

We performed power calculations to assess the required number of patients in each group. The estimated effect size was based on the study by Madsen *et al* [18]. In their study, the mean difference in hyperemic MBF between AF and SR patients was 0.70 mL/g/min in patients without CAD and 0.42 mL/g/min in patients with CAD. Because our patient population included those with and without obstructive CAD, we employed an effect size of 0.56 mL/g/min, which represents an average between these two patient groups. According to the power calculations, 28 AF patients and 111 SR patients would be needed to achieve a statistical power greater than 80% with a two-tailed *α*-level less than 0.05. Therefore, propensity score matching was performed using a 1:4 ratio, i.e., each AF patient was matched with four SR patients. Matching was performed with R 4.3.2 (R Foundation for Statistical Computing, Vienna, Austria) using a logistic regression model. AF and SR patients were matched according to the closest propensity score (within ± 0.2 standard deviations).

Apart from propensity score matching, statistical analyses were conducted using IBM SPSS Statistics version 29 (IBM, Armonk, New York, USA). We used the Shapiro-Wilk test to assess the normality of distribution for continuous variables. The results were presented as mean ± standard deviation for normally distributed data, median (interquartile range, IQR) for nonnormally distributed data, or n (%) for categorical data. Finnish Social and Health Data Permit Authority restricts the presentation of data in registry studies. Thus, we were unable to present categorical data with n < 3 in exact numbers.

To compare continuous variables between groups, we used the Student’s T-test for normally distributed data and the Mann-Whitney U-test for nonnormally distributed data. Depending on the expected cell count, either the chi-squared test or Fisher’s exact test was used to compare nominal data between groups. Statistical significance was set at p-value < 0.05.

To further assess the possible independent association between AF and hyperemic MBF, we performed multivariate general linear model analyses with three models. In each model, the presence of AF was a fixed factor. In the first model, the covariates included each variable used in propensity score matching (i.e., age, sex, body mass index, number of traditional risk factors for CAD [0–5], calcium score, and total CCTA status). In the second model, the covariates included those of the first model as well as hemoglobin and the use of beta-blockers (i.e., patient characteristics that differed statistically significantly between the patients with AF and SR and were statistically significantly associated with global hyperemic MBF). In the third model, the covariates included those of the second model as well as diastolic blood pressure after [^15^O]H_2_O bolus, glomerular filtration rate (GFR), and the use of diuretics (i.e., variables that differed statistically significantly between AF and SR patients, but which were not statistically significantly associated with global hyperemic MBF). In the multivariate general linear model analyses, we observed no unequal variances (i.e., p-value in Levene’s test was consistently ≥ 0.05) or nonnormally distributed residuals. Also, we found no significant multicollinearity between the covariates, i.e., the variance inflation factor was consistently < 2.5 [27,28].

## Results

### Clinical characteristics

Thirty-one AF patients were matched with 120 SR patients. Two of the AF patients could be matched with only two SR patients within the specified propensity score range (± 0.2 standard deviations). The patients’ clinical characteristics are shown in Table 1. Most AF patients had persistent or permanent AF. Among SR patients, 16 (13%) had previously documented AF paroxysms but had ongoing SR during PET MPI. No statistically significant differences existed between the groups in terms of age, sex, or body mass index. Also, no differences between the groups were observed in the five traditional risk factors for CAD. The use of an angiotensin-converting enzyme (ACE) inhibitor or an angiotensin II receptor (AR) blocker, a calcium channel blocker, and cholesterol medication were similar between the AF and SR patients. Beta-blocker and diuretic medications were more common in AF patients than in SR patients. Each of the three blood test results differed between the groups: GFR was lower, whereas hemoglobin and N-terminal pro-B-type natriuretic peptide (NT-proBNP) were higher in AF patients than in SR patients.

**Table 1:** Patient characteristics.

	AF patients (n = 31)	SR patients (n = 120)	p-value
**General characteristics**			
Age (years)	72 (68–74)	72 (68–76)	0.541
Females	8 (26%)	36 (30%)	0.647
Body mass index (kg/m^2^)	30.1 (26.9–33.9)	29.4 (25.1–33.5)	0.632
**Atrial fibrillation**			
Paroxysmal	5 (16%)	16 (13%)	0.771
Persistent or permanent	26 (84%)	–	–
**Symptom**			
Typical angina	3 (10%)	22 (18%)	0.248
Atypical angina	7 (23%)	20 (17%)	0.444
Dyspnea	14 (45%)	42 (35%)	0.296
Other	7 (23%)	36 (30%)	0.981
**Risk factor for CAD**			
Hypertension	28 (90%)	96 (80%)	0.181
Dyslipidemia	23 (74%)	98 (82%)	0.353
Diabetes (type 1 or type 2)	6 (19%)	22 (18%)	0.896
Ever-smoker	10 (32%)	39 (33%)	0.980
Family history of CAD	17 (55%)	72 (60%)	0.603
**Comorbidity**			
Obstructive sleep apnea	5 (16%)	22 (18%)	0.775
Systemic cancer treatment or irradiation of the chest	< 3	6 (5%)	–
Chronic obstructive pulmonary disease or interstitial lung disease	3 (10%)	6 (5%)	0.391
**Blood test results**			
GFR (mL/min/1.73 m^2^)	70 (60–79)	81 (68–89)	0.016
Hemoglobin (g/L)	151 ± 15 (n = 25)	143 ± 12 (n = 98)	0.009
NT-proBNP (ng/L)	843 (466–1,653) (n = 14)	88 (45–336) (n = 40)	< 0.001
**Medication**			
ACE inhibitor/AR blocker	22 (71%)	74 (62%)	0.337
Beta-blocker	23 (74%)	47 (39%)	< 0.001
Calcium channel blocker	9 (29%)	32 (27%)	0.792
Diuretic	13 (42%)	24 (20%)	0.011
Cholesterol medication	17 (55%)	85 (71%)	0.090
Long-acting nitrate	< 3	8 (7%)	–
Insulin	< 3	< 3	–
Other diabetes medication	4 (13%)	19 (16%)	0.787

Values are mean ± standard deviation, median (interquartile range), or n (%). ACE = angiotensin-converting enzyme, AF = atrial fibrillation, AR = angiotensin II receptor, CAD = coronary artery disease, GFR = glomerular filtration rate, NT-proBNP = N-terminal pro-B-type natriuretic peptide, SR = sinus rhythm.

### Coronary computed tomography angiography

Table 2 presents the CCTA results. Premedication with beta-blockers was equally common between AF and SR patients. Calcium score and total CCTA status showed no difference between the groups. Also, no between-group differences were observed in the status of any individual epicardial coronary artery.

**Table 2:** CCTA results.

	AF patients (n = 31)	SR patients (n = 120)	p-value
Premedication with a beta-blocker	21 (68%)	85 (71%)	0.877
Calcium score	465 (80–1,079)	274 (113–792)	0.579
Total CCTA status ≥ 3	27 (87%)	110 (92%)	0.487
LM or proximal LAD status ≥ 3	21 (68%)	73 (61%)	0.479
Distal LAD status ≥ 3	17 (55%)	66 (55%)	0.987
LCX status ≥ 3	10 (32%)	51 (43%)	0.300
RCA status ≥ 3	14 (45%)	54 (45%)	0.987

Values are median (interquartile range) or n (%). AF = atrial fibrillation, CCTA = coronary computed tomography angiography, LAD = left anterior descending coronary artery, LCX = left circumflex coronary artery, LM = left main coronary artery, RCA = right coronary artery, SR = sinus rhythm.

### Myocardial perfusion imaging

The hemodynamic parameters during PET MPI are shown in Table 3. At rest, systolic BP was similar between the groups, while diastolic BP, HR, and RPP were higher in AF patients than in SR patients. After the [^15^O]H_2_O bolus, systolic BP, HR, and RPP were similar between the groups, whereas compared to SR patients, those with AF had higher diastolic BP.

**Table 3:** Hemodynamics during PET MPI.

	AF patients (n = 31)	SR patients (n = 120)	p-value
Systolic BP at rest (mmHg)	129 (117–141)	133 (119–142)	0.812
Diastolic BP at rest (mmHg)	79 (72–84)	65 (57–75)	< 0.001
HR at rest (beats per min)	74 (63–82)	60 (54–66)	< 0.001
RPP at rest	9,840 (8,125–12,000)	7,680 (6,760–9,135)	< 0.001
Systolic BP after [^15^O]H_2_O bolus (mmHg)	124 (114–145)	126 (113–142)	0.959
Diastolic BP after [^15^O]H_2_O bolus (mmHg)	70 (65–80)	60 (52–66)	< 0.001
HR after [^15^O]H_2_O bolus (beats per min)	73 (64–88)	74 (68–86)	0.778
RPP after [^15^O]H_2_O bolus	9,513 (7,995–11,431)	9,522 (7,884–11,397)	0.833

Values are median (interquartile range). AF = atrial fibrillation, BP = blood pressure, HR = heart rate, MPI = myocardial perfusion imaging, PET = positron emission tomography, RPP = rate pressure product, SR = sinus rhythm.

Fig 2 presents the results of PET MPI. Global hyperemic MBF was lower in AF patients than in SR patients (median: 1.9 mL/g/min, IQR: 1.4–2.6 mL/g/min vs. 3.0 mL/g/min, IQR: 2.4–3.6 mL/g/min, p < 0.001). Also, hyperemic MBF in each standardized perfusion territory was lower in AF patients (p < 0.001 for each territory). Hyperemic MBF was impaired (< 2.3 mL/g/min in ≥ 1 segment) more often in AF patients (n = 25/31, 81%) than in SR patients (n = 50/120, 42%, p < 0.001). In the multivariate analyses, AF was independently associated with reduced hyperemic MBF in all three models (p < 0.001).

**Fig 2:**
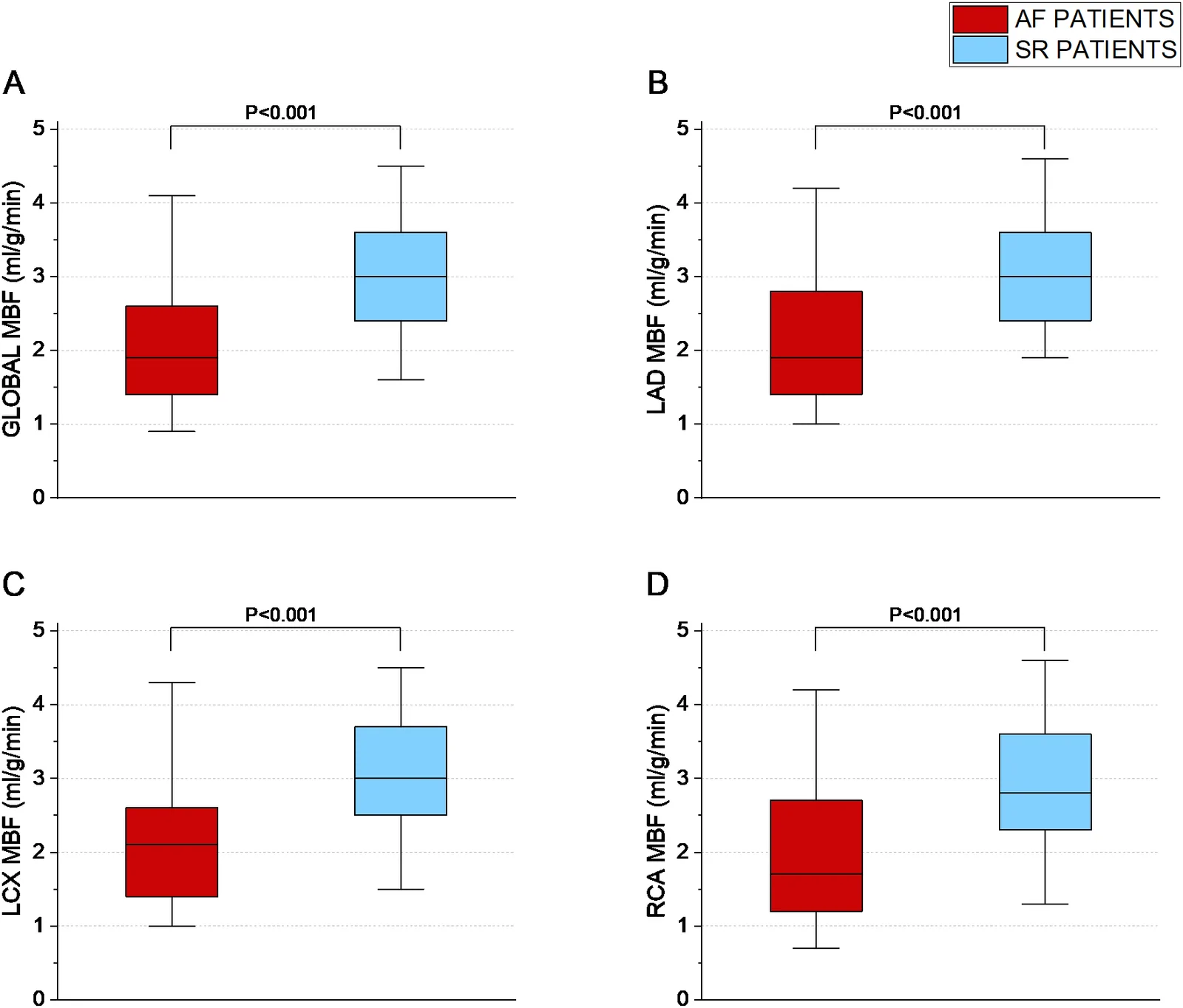
Hyperemic MBF results. The boxes indicate median and interquartile ranges. The ends of whiskers indicate 5 and 95 percentiles. AF = atrial fibrillation, SR = sinus rhythm, LAD = left anterior descending coronary artery, LCX = left circumflex coronary artery, MBF = myocardial blood flow, RCA = right coronary artery.

### Invasive coronary angiography and revascularization

Of the 25 AF patients with impaired hyperemic MBF, 18 (72%) underwent ICA. Of the 50 SR patients with MBF impairment, ICA was performed on 35 (70%). The referral rate to ICA was similar between the groups (p = 0.858). However, the revascularization frequency was significantly lower in AF patients compared to SR patients: revascularization was performed on 6/18 (33%) AF patients versus 22/35 (63%) SR patients (p = 0.041). The remaining 12/18 (67%) AF patients and 13/35 (37%) SR patients had no significant stenoses in ICA and underwent no revascularization.

Among the AF patients referred to ICA, we found no statistically significant difference in global hyperemic MBF between those revascularized and those not (1.4 ± 0.4 mL/g/min vs. 1.7 ± 0.6 mL/g/min, p = 0.070). Also, compared to revascularized AF patients, those not revascularized showed no difference in the lowest hyperemic MBF of any individual segment (mean 1.0 ± 0.3 mL/g/min vs. 1.3 ± 0.5 mL/g/min, p = 0.071).

## Discussion

Our results suggest that in symptomatic patients with suspected obstructive CAD, AF is independently associated with impaired myocardial perfusion. Myocardial perfusion was assessed using the reference standard method for MBF quantification, [^15^O]H_2_O PET MPI. The conclusion of the independent association leans against the group comparison based on detailed propensity score matching as well as the multivariate analyses with three comprehensive models.

Prior studies have demonstrated that, compared to patients with SR, those with AF have lower myocardial perfusion. In the landmark study by Range *et al*., AF patients had not only lower hyperemic MBF but also lower resting MBF and MBF under cold-pressor testing when compared with matched SR patients [13]. In addition, Wijesurendra *et al*., using magnetic resonance imaging, and Takafuji *et al*., using CT imaging to measure hyperemic MBF, reported similar results [15,17]. Our findings regarding hyperemic MBF are in line with these previous studies. To the best of our knowledge, our study is the first to include CCTA findings as matching criteria, however. Considering the confounding effect of coronary atherosclerosis is critical, as CAD and AF are interdependent, and our patients underwent coronary imaging specifically due to suspicion of obstructive CAD. Our results consistently demonstrate that AF is independently associated with reduced hyperemic MBF in this patient population.

The pathophysiology between AF and myocardial perfusion is complex, and whether this arrhythmia directly impairs perfusion remains debatable. Our study aimed to investigate the presence of an independent association between AF and myocardial perfusion. Being cross-sectional, this study lacked data to reliably assess causal mechanisms that connect the two. The direct effect of AF on myocardial perfusion could be related to irregular HR, leading to shortened and irregular duration of diastole [12,29]. AF directly affecting myocardial perfusion was supported by the finding of Takafuji *et al*. since they demonstrated an improvement in AF patients’ hyperemic MBF after catheter ablation [17]. In contrast, Wijesurendra *et al*. reported that catheter ablation yielded no significant improvement in hyperemic MBF in AF patients [15]. This discrepancy indicates that AF patients also have an underlying condition affecting myocardial perfusion. One such factor could be endothelial dysfunction [12], associated with reduced hyperemic MBF and increasing the risk of subsequent AF [30,31]. Also, in patients with hypertrophic and dilated cardiomyopathy, the presence of AF has been associated with reduced hyperemic MBF, suggesting impaired coronary microvascular function [14,32]. Overall, AF and myocardial perfusion are most likely connected by several mechanisms.

In [^15^O]H_2_O PET MPI, absolute hyperemic MBF < 2.3 mL/g/min has been established as the optimal cutoff value for detecting functionally significant CAD [24]. Accordingly, impairment in absolute hyperemic MBF most strongly guides a referral to ICA [33]. Still, in our study, only a few AF patients with hyperemic MBF < 2.3 mL/g/min had significant stenoses in ICA and were evaluated to benefit from revascularization. Moreover, Madsen *et al*. reported that only a few AF patients, even those with severely impaired hyperemic MBF, underwent revascularization [18]. Based on the results of our study and those of Madsen *et al*., we propose that for guiding a referral to ICA, AF patients would benefit from more individualized MBF cutoff values. Also, in addition to absolute MBF evaluation, the accuracy of [^15^O]H_2_O PET MPI in AF patients might be enhanced by assessing relative perfusion. This approach is supported by Madsen *et al*.‘s findings, where they observed that nearly all patients who underwent revascularization demonstrated regional hypoperfusion when evaluated using relative flow reserve measurements [18]. However, regional hypoperfusion was not a major determinant for a referral to ICA. Unfortunately, our study included only a few AF patients who underwent ICA. Thus, it lacked statistical power to assess whether impaired relative perfusion predicts revascularization in AF patients. Further research is warranted to conclude whether adjusted cutoff values for hyperemic MBF would increase diagnostic accuracy and whether relative perfusion metrics have additional value in this patient group.

The patient groups showed differences in each three blood test results, that is, GFR, hemoglobin, and NT-proBNP. The possible confounding effect of GFR and hemoglobin on global hyperemic MBF was considered with the multivariate analyses. When both measurements were included in the multivariate models, AF remained independently associated with global hyperemic MBF. NT-proBNP was higher in AF patients compared to SR patients. However, given that the measurement was available in only 36% (54/151) of the included patients, we excluded it from the multivariate analyses. In AF patients, interpreting elevated NT-proBNP is challenging. Jones *et al*. recently showed that in 155,347 primary care patients, NT-proBNP was 724 (IQR 245–1,535) ng/L in patients with AF but without diagnosed heart failure and 111 (IQR 51–250) ng/L in patients without AF but with diagnosed heart failure [34]. These figures closely resemble those in our study and highlight how the presence of AF is associated with significantly elevated NT-proBNP levels even in the absence of heart failure. Furthermore, several studies have demonstrated that AF is independently associated with significantly elevated NT-proBNP, as Liu *et al*. showed in their meta-analysis [35]. Since patients with diagnosed heart failure and cardiomyopathies, as well as those with prior myocardial infarction were excluded from our study, we suggest that AF presence mostly explains the differing NT-proBNP levels. Still, we acknowledge that recent echocardiography results were unavailable for most patients. We were thereby unable to evaluate the echocardiography-based measures of cardiac structure and function.

CCTA and ICA showed some discrepancies. While most patients had suspected significant stenosis in CCTA, only 33% of AF patients and 63% of SR patients showed a significant stenosis in ICA and were subsequently revascularized. In line with the latest practical CCTA guideline, ≥ 50% stenosis was used as the cutoff value that prompted consecutive PET MPI [20]. Still, angiographic stenosis severity correlates poorly with the lesion’s functional significance even up to 90% stenosis [36]. Also, compared to ICA, the specificity of CCTA to detect functionally significant CAD is lower [4]. Based on PET MPI, the decision to refer a patient to ICA was guided by hyperemic MBF of < 2.3 mL/g/min even when AF was present. The lower specificity of CCTA compared to ICA, and the assumably suboptimal hyperemic MBF cutoff value for AF patients likely explain the significantly lower revascularization frequency of AF patients. This highlights the demand for more individualized MBF cutoff values even when applying a hybrid imaging approach.

We acknowledge that this single-center study had limitations. Unfortunately, resting MBF was unavailable for most patients, since the standard PET MPI protocol at Kuopio University Hospital includes only hyperemic imaging. Therefore, we were also unable to determine myocardial flow reserve, i.e., the ratio of hyperemic MBF to resting MBF. The higher resting RPP of AF patients compared to SR patients indicates that in AF patients, the demand for resting MBF is higher, which would also affect myocardial flow reserve. Due to the lack myocardial flow reserve assessment, we could not evaluate the vasodilatory capacity of the patients. The number of AF patients was limited. However, our initial patient population comprised 874 patients, which allowed us to perform propensity score matching with a 1:4 ratio. After appropriate statistical power calculations, we included 151 patients in the final analyses. Females were rather underrepresented in this study, which may affect the generalizability of our findings. Some measurements may have suffered from lower reliability in AF patients compared to SR patients. For example, automated blood pressure measurement is generally less accurate during uneven cardiac rhythm [37]. In addition, ongoing AF may introduce some unreliability in MBF quantification. However, the determination of MBF was based on imaging in which the shortest frames were 5 seconds long. In 5-second frames, variation caused by beat-to-beat hemodynamic fluctuations is already significantly attenuated. Moreover, a time-activity curve—upon which the calculation was based—was fitted to data comprising multiple frames. In this overall process, the impact of variations caused by instantaneous beat-to-beat fluctuations was further reduced. Consequently, the potential source of error arising from the irregular heart rhythm of AF patients is unlikely to be significant. Additionally, we aimed to minimize the contribution of MBF quantification errors by excluding patients with technically unreliable PET MPI such as those who demonstrated significant motion artefacts during imaging. Finally, since this study was cross-sectional, we were unable to assess the possible causality between AF and myocardial perfusion.

## Conclusions

In patients with symptoms suggestive of obstructive CAD, the presence of AF was independently associated with impaired hyperemic MBF. Clinicians should consider this association when interpreting PET MPI results as part of individualized clinical decision-making for this highly prevalent patient group. Further research is warranted to establish whether patients with AF would benefit from adjusted hyperemic MBF cutoff values to more accurately diagnose obstructive CAD.
